# Compensation for metabolic dietitians practicing in the United States: 2023 genetic metabolic dietitians international professional status survey

**DOI:** 10.1016/j.ymgmr.2024.101147

**Published:** 2024-09-29

**Authors:** Krista Viau, Sommer Gaughan, Jessica Kopesky, Beth Ogata, Heather Saavedra, Mary Sowa, Erin MacLeod

**Affiliations:** a Genetics and Genomics, Boston Children's Hospital, Boston, MA, USA; b Clinical Genetics and Metabolism, Children's Hospital Colorado, Aurora, CO, USA; c Department of Clinical Nutrition, Children's Wisconsin, Milwaukee, WI, USA; d Department of Pediatrics, University of Washington, Seattle, WA, USA; e Division of Medical Genetics, Department of Pediatrics, McGovern Medical School at the University of Texas Health Science Center at Houston (UTHealth Houston), Children's Memorial Hermann Hospital, Houston, TX, USA; f Clinical Nutrition and Lactation, CHOC Children's, Orange, CA, USA; g Rare Disease Institute, Children's National Hospital, Washington, DC, USA

**Keywords:** Metabolic dietitian, Salary, Compensation, Benefits, Job satisfaction, Workforce

## Abstract

**Background:**

Genetic Metabolic Dietitians International (GMDI) conducted a professional status survey of metabolic dietitians working in the United States to describe job satisfaction and establish salary and compensation benchmarks specific to metabolic dietitians.

**Methods:**

The survey was anonymously administered in a web-based format via REDCap between October and November 2023. Registered dietitians working with inborn errors of metabolism (IEM) were eligible to participate.

**Results:**

A total of 178 surveys were received and 147 were included in the final analysis. Most respondents were female (96 %), worked in a clinical setting (83 %), and held a graduate degree (75 %), while 30 % had one or more board certifications and 8 % were faculty. Respondents specialized in genetic metabolic nutrition for a median of 6 years (IQR 2–15). Overall satisfaction with one's professional focus in IEM was high among respondents (87 %), though 40 % reported dissatisfaction with earnings potential in their current position.

The median annual, full-time salary for US-based metabolic dietitians in all work settings was $80,400 (IQR $67,100-96,000). After excluding dietitians working in the pharmaceutical and/or nutrition industry (*n* = 14), the median annual salary decreased to $76,200 (IQR $66,700-91,000). Increased years' experience, responsibility (e.g., supervisor), expertise (i.e., board certification), and categorization as a Level II dietitian or higher were associated with higher annual salary.

**Conclusion:**

The results of the 2023 GMDI Professional Status Survey provide insight into the current compensation and benefits of metabolic dietitians practicing in the US. These data can be used to support individual efforts to secure equitable compensation for the metabolic dietitian's critical role in the medical nutrition therapy for individuals with IEM.

## 1 Introduction

Medical nutrition therapy remains the primary treatment to prevent or delay the long-term consequences of many inborn errors of metabolism (IEM) despite ongoing therapeutic advancements in the field. The nutrition management of IEMs is complex and requires an individualized and closely monitored diet plan to treat the underlying IEM while providing nutrition for optimal growth and development. The results of inadequate or inappropriate nutrition intervention are often severe. Depending on the condition, it can result in frequent hospitalizations, severe cognitive impairment, multi-organ failure, cerebral edema, or even death.

In the United States, medical nutrition therapy for individuals with IEMs is provided by registered dietitians (RDs) who specialize in this field. To become a registered dietitian, one must earn a Bachelor's degree in dietetics, complete an accredited internship, and, as of January 1, 2024, earn a Master's degree before being eligible to take the registration exam for dietitians.

Despite the highly specialized nature of genetic metabolic nutrition, metabolic dietitians rarely receive much, if any, formal education specific to the management of IEM. This gap necessitates significant self-study and on-the-job training to achieve competency. To address this need, Genetic Metabolic Dietitians International (GMDI) was founded in 2005. This professional organization of metabolic dietitians and healthcare practitioners specializes in IEM, with a primary mission to provide standards of excellence and leadership in nutrition therapy for genetic metabolic disorders through clinical practice, education, advocacy, and research. Over the past two decades, educational opportunities have expanded significantly [1] and professional competencies have been developed [2]. Following a recent needs assessment, GMDI launched a new metabolic mentorship program [3]. However, there is no board certification specific to genetic metabolic nutrition as a tool to demonstrate one's expertise and potentially serve as a path towards career advancement.

Due, in part, to the rarity of IEMs and multifaceted management approach, metabolic dietitians in a clinical setting perform a wide range of job functions. In addition to direct clinical care, metabolic dietitians often serve as the first point of contact for their patients and function as case managers and project managers to meet the complex needs of this population. Furthermore, due to the critical role of medical nutrition therapy during acute illness in many metabolic disorders, metabolic dietitians are frequently required to be available during non-working hours or to be “on call.”

Retaining experienced metabolic dietitians is essential to ensure optimal patient care and mentor the next generation of professionals. GMDI conducts a Professional Status Survey (PSS) of metabolic dietitians approximately every four years. In 2020, nearly 25 % of PSS respondents (*n* = 28 out of 125) indicated they were moderately to very likely to seek alternative employment within the next five years (unpublished data). The primary reasons cited were limited earnings potential and burnout. Similarly, a 2023 National Coordinating Center Genetics Workforce Survey, which did not explore salary, found that 40 % of metabolic dietitian respondents (*n* = 54 out of 133) reported experiencing symptoms of burnout [4].

Here we present the results of the fifth GMDI PSS of metabolic dietitians, including GMDI members and non-members working in the United States, to establish salary and compensation benchmarks specific to metabolic dietitians and describe job satisfaction.

## 2 Materials and methods

The survey was anonymously administered in a web-based format via REDCap between October 15, 2023 and November 22, 2023. A full copy of the questionnaire is available as supplementary material. Participants were recruited via email advertisements to GMDI members and on a listserv for metabolic dietitians (PNO-METLAB listserv through Emory University), which includes both GMDI members and non-members. Only registered dietitians who were currently working with IEM were eligible to participate. While dietitians working internationally were eligible, the results presented here reflect only metabolic dietitians working in the United States.

Annual, full-time salary was determined from the number of hours per week respondents were assigned to work and the corresponding annual gross salary. For respondents who reported working fewer than 40 h per week, annual salaries were normalized to reflect a full-time position (i.e., 40 h * 52 weeks or 2080 h/year).

To evaluate the impact of cost-of-living (CoL) on annual salary, each state and the District of Columbia (DC) were categorized into quintiles based on the CoL ranking from Forbes Advisor (Cost Of Living By State Statistics & Trends In 2023). This ranking was generated based on yearly expenses for housing, healthcare, taxes, food, and transportation.

Data were descriptively analyzed using Stata/IC 18.0. Continuous variables were reported as median (interquartile range (IQR: 25th–75th percentile)), and categorical variables as frequencies and proportions. To maintain anonymity, only median salary data were reported for subgroups with 5–9 respondents (IQR marked “ID” for insufficient data). Subgroups with fewer than five respondents were not included. Bivariate analyses were conducted to assess potential relationships with respondents' salary. Wilcoxon rank sum test was used for binary variables, Kruskal Wallis test for categorical variables, and Spearman rank correlation coefficients for continuous variables.

## 3 Results and discussion

**Fig. 1:**
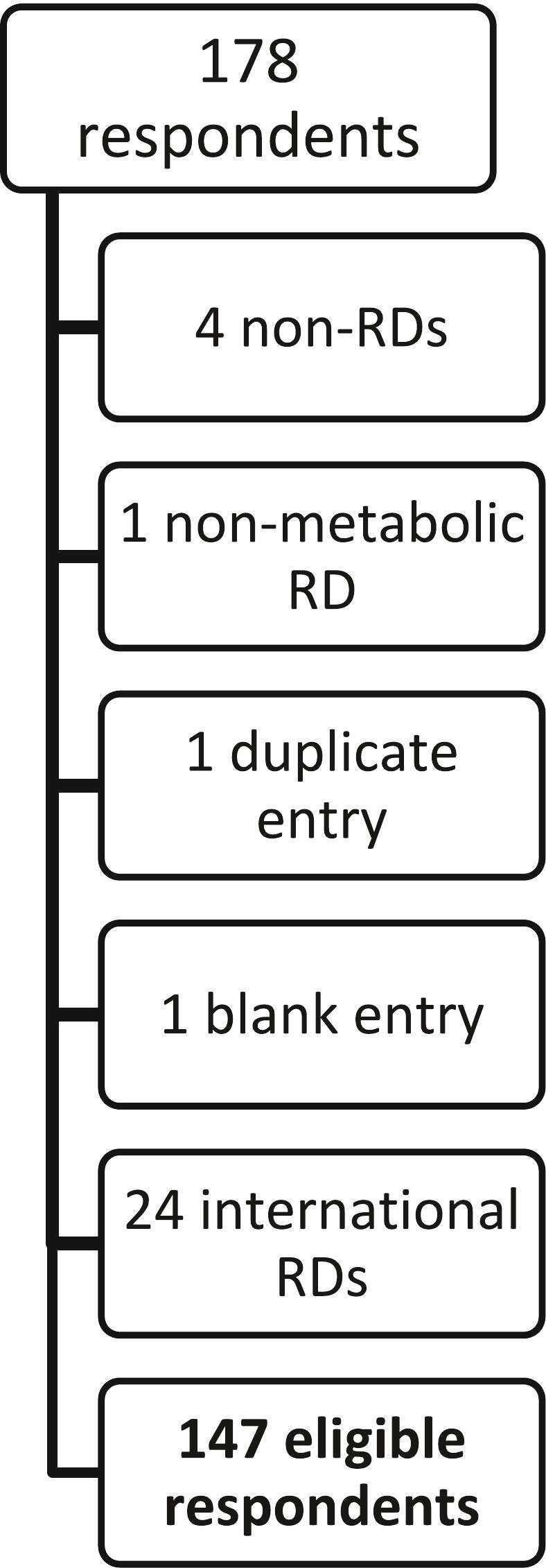
Respondent flow chart.

A total of 178 surveys were received and 147 were included in the final analysis (Fig. 1). It was not feasible to calculate a precise response rate based on the recruitment methods. However, we estimate the response rate was less than 50 %, as there are 289 US-based metabolic dietitians who are current GMDI members.

### 3.1 Demographics and work setting

Most respondents were female (96 %), worked in a clinical setting (83 %), held a graduate degree (75 %), and were assigned to work solely with IEM (73 %; Table 1). Respondents specialized in genetic metabolic nutrition for a median of 6 years (IQR 2–15), ranging from 0.2 to 44 years, and had a median of 3 years' (IQR 0–7) experience as a RD before working with IEM. Despite most respondents working in a clinical setting, 61 % reported spending ≥25 % of their time working remotely (Table 1).

**Table 1:** Respondent characteristics and work setting.

	Respondents n (%)
Years' Experience as a Registered Dietitian	
< 5 years	29 (20)
5–9 years	33 (23)
10–19 years	45 (32)
≥ 20 years	36 (25)

Highest Educational Degree	
Bachelor's degree	36 (25)
Master's degree	102 (69)
Doctoral degree	8 (6)
One or more Board Certification(s)	43 (30)

Current Work Setting	
University medical center	65 (47)
Public or private hospital	49 (36)
Nutrition/pharmaceutical industry	14 (10)
University (non-medical)	3 (2)
Other (e.g., government, self-employed)	7 (5)

Employer⁎	
Hospital - Nutrition Department/Division	39 (34)
Hospital - Genetics Department/Division	31 (27)
University - Nutrition Department/Division	4 (3)
University - Genetics Department/Division	33 (29)
Both Nutrition & Genetics	6 (5)
Other	2 (1)

Percentage of Time Assigned to IEM	
< 50 %	9 (7)
50–99 %	27 (20)
100 %	99 (73)

Percentage of Time Working Remotely	
< 25 %	54 (39)
25–49 %	23 (17)
50–74 %	28 (20)
75–100 %	34 (24)
Faculty Position	15 (8)
Tenure track	4 (3)
Supervisor (over 1–16 employees)	23 (17)

⁎ Respondents working in hospital and/or university settings.

#### 3.1.1 Dietitians working in a clinical setting

Only 60 % of respondents working in a clinical setting billed for their services. Clinical nutrition services were billed under the dietitian and physician (22 %), as part of a comprehensive fee for an interdisciplinary visit (15 %), under the dietitian only (14 %), or as a facility fee (8 %). Metabolic dietitians' salaries were paid from one or more of the following funds: hospital/university fund(s) (*n* = 87), newborn screening grant(s) (*n* = 33), research grant(s) (*n* = 13), and other grant(s) (*n* = 7).

Forty-six percent of clinical, metabolic dietitians (*n* = 51) reported their current employer used a dietitian career ladder. These respondents were categorized as a Level I (n = 8), Level II (*n* = 17), Level III (*n* = 15), or Level IV/Advanced Practice (*n* = 9) dietitian. When compared to Level I dietitians, Level II, Level III, and Level IV dietitians had more years' experience as a RD and more years working for their current employer (Supplementary Table 1). Level II, III, and IV dietitians also had a greater proportion of respondents with a board certification compared to Level I dietitians. The proportion of respondents with a graduate degree was similar across dietitian levels (ranging from 71 to 89 %), which may, in part, reflect the new minimum educational requirement of a Master's degree for prospective dietitians.

#### 3.1.2 Dietitians working in industry

Fourteen respondents worked for a nutrition or pharmaceutical company for a median of 4 years (IQR 2–12), ranging from 1 to 16 years. Of these respondents, eight worked in medical affairs, two in sales, two in research and development, and two in patient support. Prior to transitioning to an industry position, 36 % (*n* = 5) had over 10 years' experience working clinically with IEM, 50 % (*n* = 7) had between 1 and 10 years' experience, and 14 % (*n* = 2) had no clinical experience. The primary reason(s) for transitioning from a clinical to an industry position included increased compensation (*n* = 6), professional development opportunities (n = 5), burnout and stress from clinical position (*n* = 4), and/or flexible and/or remote work schedule (*n* = 3).

### 3.2 Annual salary

Most respondents worked full time (76 %) and were salaried (89 %) with 10 % paid hourly and 1 % in private practice. Among respondents working part-time (*n* = 33), 27 % (*n* = 9) were hourly (non-exempt) or private practice employees. Respondents' annual full-time salary is detailed in Table 2. Considering the salary of dietitians working in industry was approximately two-fold that of non-industry dietitians, subgroup analyses reflect only non-industry metabolic dietitians unless otherwise specified.

**Table 2:** Annual, full-time salary for metabolic dietitians practicing in the United States*.

		Percentile				
	n	10th	25th	50th	75th	90th
All Respondents	132	61,000	67,100	80,400	96,000	137,000
Non-Industry RDs	121	61,000	66,700	76,200	91,000	112,000

Years as a Registered Dietitian						
< 5 years	26	55,000	59,000	63,000	72,000	76,000
5–9 years	28	62,000	66,700	72,900	86,700	112,000
10–19 years	36	65,000	74,200	83,400	92,300	113,500
20–29 years	14	73,600	75,600	88,800	104,200	108,300
≥ 30 years	15	72,800	83,600	90,000	120,000	137,000

Highest Educational Degree						
Bachelor's degree	28	62,000	66,200	80,000	90,900	104,400
Master's degree	85	60,300	66,100	75,100	90,000	110,000
Doctorate degree	7	ID	ID	106,100	ID	ID

Work Setting						
University Medical Center	63	62,000	66,000	75,000	91,000	113,500
Public Hospital	25	60,000	65,000	75,000	85,300	102,000
Private Hospital	22	59,900	67,200	82,400	93,600	106,100
Nutrition and/or Pharmaceutical Industry	11	123,000	123,000	152,000	175,000	198,000

Dietitian Career Ladder						
No career ladder at facility	63	60,000	65,000	73,600	86,300	98,000
RD level I	8	ID	ID	61,100	ID	ID
RD level II	17	65,000	73,000	85,000	92,500	108,300
RD level III	15	75,000	80,000	85,000	120,000	147,100
RD level IV or advanced practice	9	ID	ID	95,500	ID	ID

Specialty Board Certification(s)						
No	80	60,000	64,100	73,600	87,100	109,200
Yes	40	72,400	76,800	85,200	95,700	132,000

Faculty Position						
No	105	61,000	66,000	75,100	90,000	104,400
Yes	14	60,000	78,000	97,600	119,000	190,000
Instructor/Lecturer	6	ID	ID	76,400	ID	ID
Assistant or Associate Professor	6	ID	ID	114,500	ID	ID

Supervisory Role						
No supervisory role	99	60,000	65,000	75,000	86,600	100,000
Supervisory role	20	73,500	80,000	98,700	119,500	171,800

Cost of Living⁎⁎						
1 - AK, CA, CT, DC, HI, MA, MD, NJ, NY, OR, WA	32	75,100	87,900	96,900	119,500	147,100
2 - CO, DE, IL, MN, NH, NV, RI, UT, VA, VT	15	61,000	64,100	68,700	86,300	96,200
3 - AZ, FL, GA, ID, ME, NE, PA, TX, WI, WY	34	60,000	66,100	76,100	85,500	91,000
4 - IA, IN, KY, LA, MI, MT, NC, ND, OH, SD	18	57,900	60,000	64,400	82,000	85,000
5 - AL, AR, KS, MO, MS, NM, OK, SC, TN, WV	18	65,000	71,500	73,200	80,000	95,500

RD, Registered Dietitian; ID, insufficient data.

*Reported annual salaries reflecting <40 h/week were normalized to reflect a full-time position (40 h * 52 weeks or 2080 h/year) and rounded to the nearest $100. Unless otherwise specified, salary data reflect non-industry metabolic dietitians.

⁎⁎ CoL ranking by state as reported by Forbes Advisor (Cost Of Living By State Statistics & Trends In 2023).

The median annual, full-time salary for US-based metabolic dietitians in all work settings was $80,400 (IQR $67,100-96,000). After excluding dietitians working in industry, the median annual salary was $76,200 (IQR $66,700-91,000). These results demonstrate an increase from the 2020 median salary for US-based metabolic dietitians (for both all work settings and non-industry only), which was $70,000 (GMDI's 2020 PSS; unpublished data). The median salary is comparable to the most recent Academy of Nutrition and Dietetics Compensation and Benefits Survey of Dietetics Profession for practicing RDs up to January 1, 2024, which showed the median hourly wage for RDs in all positions was $37.98 per hour, which equates to an annual, full-time salary of approximately $79,000 [5].

Compensation for metabolic dietitians can also be compared to related disciplines, such as genetic counselors. Genetic counselors often work in metabolic clinics to help interpret genetic testing results and provide counseling for patients seeking information about how inherited conditions might affect them or their families. The National Society of Genetic Counselors reported, as of December 31, 2022, the median annual, full-time salary for genetic counselors was $87,000 for those with direct patient care positions, $125,000 for non-direct patient care positions, and $104,005 for mixed positions [6]. Thus, median salary for non-industry metabolic dietitians is approximately $10,000 less per year compared to genetic counselors in direct patient care positions, despite similar responsibilities and training requirements.

Similarly, as of May 2023, Registered Nurses (RNs) earned a median annual wage of $86,070 [7]. While nursing encompasses a wide range of specialties, metabolic dietitians often perform case management roles similar to those of case management RNs, particularly for complicated patients. The median annual salary of non-industry metabolic dietitians is approximately $10,000 less per year compared to RNs.

#### 3.2.1 Experience and responsibility

Years' experience as a RD was positively associated with median salary (rho = 0.51, *p* < 0.001) with consistent increases in 5- and 10-year increments (Table 2). However, the rate of salary increase declined with more years' experience, which may be related, in part, to reaching the top of one's salary range. Categorization as a dietitian Level II or higher corresponded to increased salary compared to both Level I dietitians and dietitians without a career ladder (p < 0.001). Increased responsibility (i.e., supervisory and faculty positions; *p* = 0.014) and specialization with a board certification (*p* = 0.007) were also associated with higher salary.

#### 3.2.2 Education

Interestingly, respondents with a Master's degree had a lower median salary than those with a Bachelor's degree, though the difference was non-significant (*p* = 0.274). As noted above, a Master's degree is the minimum educational requirement for prospective RDs as of January 2024, which could suggest the discrepancy in salary would be related to years of experience. However, years' experience as a RD was similar between respondents with a Bachelor's and Master's degree, as was the proportion of respondents with a career ladder at their facility, proportion with a supervisory position and/or responsible for managing a budget, proportion working in a private hospital, and CoL distribution (data not shown). However, a greater proportion of those with a Bachelor's degree had a board certification (42 % vs. 27 %). The higher median salary for metabolic dietitians with a Bachelor's degree is not fully explained by the available data.

#### 3.2.3 Cost-of-living

Median annual salary was significantly different based on CoL quintile (*p* < 0.001). However, only respondents living in the highest CoL quintile had a median salary greater than the national median (Table 2). CoL appeared to have no clear impact on salary for those working in the remaining 40 states. Considering many states have a single metabolic clinic, CoL was classified into quintiles based on the state of the respondent's employer to protect respondent anonymity. However, the broad categorization of CoL may have lacked the necessary precision to assess the impact of CoL on respondents' salary.

#### 3.2.4 Increase in salary

In 2023, 66 % (*n* = 79) of non-industry dietitians and 85 % (*n* = 11) of industry dietitians received an increase in annual salary/hourly wage. Of those respondents, the increase in salary was based on merit (*n* = 53), a cost-of-living adjustment (*n* = 38), market adjustment (*n* = 14), promotion (*n* = 12), and/or other reasons (*n* = 5). Overall, the median percentage increase in annual salary was 4 % (IQR 3–6 %). The largest increases were found in individuals who received a promotion (median 10 %, IQR 8–15 %). Of the 41 % (*n* = 49) of non-industry dietitians who actively attempted to increase their annual salary in 2023, 59 % were at least partially successful. Of the 38 % (n = 5) of industry dietitians who attempted to increase their annual salary in 2023, all were at least partially successful.

### 3.3 Benefits

Nearly all respondents (96 %) reported receiving a benefits package from their employer. Most respondents reported their employer offered the following: health insurance (99 %), dental insurance (98 %), vision insurance (98 %), life insurance (94 %), disability insurance (91 %), parental leave (paid or unpaid) (86 %), pre-tax reimbursement programs (73 %), wellness programs (73 %), 401 k retirement account (67 %), tuition reimbursement (66 %), 403b retirement account (58 %), and parking/transportation (52 %). However, for most, significantly fewer respondents reported that their employers contributed towards these benefits (Supplementary Table 2).

Most respondents received paid time off (PTO) for all (42 %) or some (46 %) federal holidays. Some respondents received financial compensation (15 %) or compensatory time off (14 %) for working overtime. Annual PTO and other quality of life benefits are detailed in Table 3. Most (84 %) respondents were allowed to carryover earned PTO from one year to the next.

**Table 3:** Quality of life benefits.

	Respondents n (%)
Annual paid time off (personal and sick leave)	
≤ 10 days	4 (3)
10–19 days	23 (18)
20–29 days	58 (45)
≥ 30 days	35 (27)
Unlimited/discretionary	8 (6)

Other benefits	
Paid time off for professional development	86 (67)
Flexible work schedule	67 (52)
Extended (>12 weeks) and/or paid parental leave	39 (30)
Cash bonuses	24 (19)
Additional time off (e.g., two weeks off at the end of the year)	15 (12)

Professional development opportunities are particularly important to develop competence and maintain expertise in genetic metabolic nutrition considering the limited training in most dietetic programs. Most employers provided at least partial reimbursement for professional development expenses (Table 4). The highest rates of reimbursement were for professional conference registration and travel.

**Table 4:** Employer reimbursement for professional development expenses.

	Complete n (%)	Partial n (%)	None n (%)	Unsure n (%)
Conference registration fees	71 (55)	44 (34)	10 (8)	3 (2)
Conference travel expenses	63 (49)	50 (39)	12 (9)	3 (2)
Professional membership fees	56 (44)	17 (13)	51 (40)	3 (2)
Licensure fees	52 (41)	12 (9)	62 (48)	2 (2)
Continuing education credit fees	36 (29)	41 (33)	44 (35)	5 (4)
Scientific books/journals	33 (26)	42 (33)	44 (34)	9 (7)
Board certification exams	29 (23)	23 (18)	51 (40)	24 (19)

### 3.4 Paid professional activities

In addition to their primary employment, 29 % (*n* = 43) of respondents reported participating in one or more of the following paid professional activities over the last year: consulting (*n* = 31), invited lectures (*n* = 24), private practice (n = 6), teaching (n = 5), writing (n = 1), and other (n = 7). The top two reasons for engaging in outside professional activities were additional financial compensation (*n* = 36) and professional development opportunities (n = 28).

### 3.5 Satisfaction

Overall satisfaction with one's professional focus in IEM was high among respondents with 32 % being moderately and 55 % being highly satisfied. Respondents reported the highest rates of satisfaction (rated as satisfied or extremely satisfied) for patient relations (90 %) and scientific rigor (85 %; Supplementary Table 3). The lowest rates of satisfaction were reported for earnings potential (40 % dissatisfied or extremely dissatisfied).

When asked for additional comments, metabolic dietitians described challenges relating to (1) the wide scope of responsibilities of a metabolic dietitian; (2) adequately justifying metabolic dietitian full time equivalents to management, which may be due to the time intense nutrition management required for an IEM patient population compared to other patient populations; and (3) achieving an increase in salary, which some respondents related to a lack of a board certification specific to genetic metabolic nutrition.

### 3.6 Limitations

Limitations of the current survey include potential for nonresponse bias, as the respondents reflect a subset of metabolic dietitians practicing in the US. Data for this survey were self-reported, and there may have been some variation in the respondents' interpretation of the questions. Additionally, the descriptive statistics and bivariate analyses presented here do not fully account for the numerous factors affecting compensation and benefits.

## 4 Conclusion

The results of the 2023 GMDI Professional Status Survey provide insight into the current compensation and benefits of metabolic dietitians practicing in the United States. The survey also highlighted factors associated with higher compensation, such as board certification, categorization as a Level II-IV dietitian, increased years' experience, and supervisory and/or faculty positions.

The impact of medical nutrition therapy on health outcomes of individuals with IEMs is both clear and significant. While metabolic dietitians often have responsibilities beyond IEM nutrition management, these additional job functions are not often included in job descriptions. Therefore, they are unlikely to be considered when determining compensation for metabolic dietitians. The high rates of dissatisfaction with earnings potential, salary discrepancy between metabolic dietitians and similar disciplines, and recently reported rates of burnout among metabolic dietitians are cause for concern regarding retention of these highly skilled and unique team members. Specifically, there is a high risk of clinical metabolic dietitians moving into positions in industry, project management, and/or case management.

Genetics divisions, under which more than half of respondents are directly employed, are multidisciplinary and may only contain one or two metabolic dietitians. Division leaders should take a holistic view of salaries across the division and consider the diverse roles of metabolic dietitians. In contrast, in Nutrition departments, the job description for a clinical dietitian may not appropriately describe the job functions of a metabolic dietitian. We encourage those department leaders to develop job descriptions and/or implement a dietitian career ladder that includes competencies and roles specific to metabolic dietitians.

By advocating together for better compensation and recognition, we can ensure that metabolic dietitians are adequately supported, enabling them to continue providing essential services that improve patient outcomes and reduce healthcare costs. The salary data presented here can bolster individual efforts to secure equitable compensation and advancement opportunities for the metabolic dietitian's vital role in the medical nutrition therapy for individuals with IEMs.

## CRediT authorship contribution statement

**Krista Viau:** Writing – review & editing, Writing – original draft, Methodology, Formal analysis, Conceptualization. **Sommer Gaughan:** Writing – review & editing, Methodology, Conceptualization. **Jessica Kopesky:** Writing – review & editing, Writing – original draft, Methodology, Conceptualization. **Beth Ogata:** Writing – review & editing, Methodology, Conceptualization. **Heather Saavedra:** Writing – review & editing, Methodology, Conceptualization. **Mary Sowa:** Writing – review & editing, Methodology, Conceptualization. **Erin MacLeod:** Writing – review & editing, Writing – original draft, Methodology, Formal analysis, Conceptualization.

## Declaration of competing interest

None.

## Data Availability

The data that has been used is confidential.

## Appendix A Supplementary data

Supplementary material 1 Supplementary material 1: Questionnaire
