# Supplementary material for: Compensation for metabolic dietitians practicing in the United States: 2023 genetic metabolic dietitians international professional status survey
Source: Mol Genet Metab Rep. 2024 Sep 29;41:101147. doi: 10.1016/j.ymgmr.2024.101147 (PMC11470630; doi:10.1016/j.ymgmr.2024.101147)
Supplement: Supplementary material 1 [file mmc1.pdf]

2023 GMDI Professional Status Survey

GMDI is conducting its 5th survey to address salary and benefit compensation for dietitians who participate in nutrition management of inborn errors of metabolism (IEM), including clinical and industry dietitians. This area of dietetics requires specialized practice skills that incorporate a background understanding of biochemistry, genetics and genomics. The purpose of this survey is to compile information about job responsibilities and salaries that can be used to support individual efforts to secure equitable compensation for their significant involvement in the medical intervention of patients diagnosed with these rare conditions.

The questionnaire will take approximately 20-30 minutes to complete, though you do NOT have to complete the entire questionnaire at once.

Note: We will be asking for your annual salary in US dollars. If you need to convert your salary to US dollars, please use the following website: http://www.xe.com/currencyconverter/

Thank you for your time and participation!

Demographics

Are you a Registered Dietitian (RD)?

- ◯ Yes
- ◯ No

In what country do you currently work?

- ◯ United States
- ◯ Canada
- ◯ Other

If other, please specify which country.

____

In which Genetic Region do you work?

- ◯ Region 1- New England Regional Genetics Group, including: Connecticut, Massachusetts, Maine, New Hampshire, Rhode Island and Vermont
- ◯ Region 2- New York/Mid Atlantic Regional Genetics Group, including: Delaware, District of Columbia, Maryland, New Jersey, New York, Pennsylvania, Virginia and West Virginia
- ◯ Region 3- Southeast NBS and Genetics Collaborative, including: Alabama, Florida, Georgia, Louisiana, Mississippi, North Carolina, Puerto Rico, South Carolina, Tennessee and the Virgin Islands
- ◯ Region 4- Region 4 Genetics Collaborative, including: Illinois, Indiana, Kentucky, Michigan, Minnesota, Ohio and Wisconsin
- ◯ Region 5- Heartland Genetics and Newborn Screening Collaborative, including: Arkansas, Iowa, Kansas, Missouri, North Dakota, Nebraska, Oklahoma and South Dakota
- ◯ Region 6- Mountain States Genetics Regional Collaborative Center, including: Arizona, Colorado, Montana, Nevada, New Mexico, Texas, Utah and Wyoming
- ◯ Region 7- Western States Genetic Services Collaborative, including: Alaska, California, Guam, Hawaii, Idaho, Oregon and Washington

In which state category* do you work?<br><br>*The categories are based on cost-of-living.	◯ 1- Alaska, California, Connecticut, District of Columbia, Hawaii, Maryland, Massachusetts, New Jersey, New York, Oregon, or Washington<br>◯ 2- Colorado, Delaware, Illinois, Minnesota, Nevada, New Hampshire, Rhode Island, Vermont, Virginia, Utah<br>◯ 3- Arizona, Florida, Georgia, Idaho, Maine, Nebraska, Pennsylvania, Texas, Wisconsin, Wyoming<br>◯ 4- Indiana, Iowa, Kentucky, Louisiana, Michigan, Montana, North Carolina, North Dakota, Ohio, South Dakota<br>◯ 5- Alabama, Arkansas, Kansas, Mississippi, Missouri, New Mexico, Oklahoma, South Carolina, Tennessee, West Virginia
Please describe the best term(s) that describe your gender identity.	◯ Woman<br>◯ Man<br>◯ Transgender woman<br>◯ Transgender man<br>◯ Non-binary<br>◯ None of these items describe me<br>◯ Prefer not to answer
What is your age?	◯ 20-29 years<br>◯ 30-39 years<br>◯ 40-49 years<br>◯ 50-59 years<br>◯ 60-69 years<br>◯ 70+ years

Education and Work History

What is your highest educational degree?	◯ Bachelor's degree<br>◯ Master's degree<br>◯ Doctorate<br>◯ Other
Do you currently have one or more CDR board certifications (e.g., Certified Specialist in Pediatrics) or equivalent (e.g., Certified Lactation Counselor)?<br>Please note, this does not include state licensure.	◯ Yes<br>◯ No
How many years have you been employed as a dietitian?	______________________________<br>(years)
How many years have you been working with patients diagnosed with inborn errors of metabolism (IEM)?	______________________________<br>(years)
How many years have you been with your current employer?	______________________________<br>(years)

Work Environment

What is your current job title?

____

What is your primary work setting as a dietitian?

- ◯ University Medical Center
- ◯ University (Non-Medical Center)
- ◯ Private Hospital/Medical Facility
- ◯ Public Hospital/Medical Facility
- ◯ Federal/State/County Government
- ◯ Self-employed/Private practice
- ◯ Nutrition/Pharmaceutical Industry
- ◯ Other

Which option best describes your employer?

- ◯ Hospital, Nutrition department/division
- ◯ Hospital, Genetics/Metabolism department/division
- ◯ University, Nutrition department/division
- ◯ University, Genetics/Metabolism department/division
- ◯ Combination of Nutrition and Genetics Dept/Division
- ◯ Other

If other, please specify.

____

Does your facility use a career ladder for clinical dietitians (i.e., classifying dietitians as Level I, II, III, or IV)?

- ◯ Yes
- ◯ No

If yes, how are you currently classified?

- ◯ Level I dietitian
- ◯ Level II dietitian
- ◯ Level III dietitian
- ◯ Level IV dietitian
- ◯ Advanced practice/specialist

In what area do you currently work?

- ◯ Medical Affairs
- ◯ Research and Development
- ◯ Sales
- ◯ Marketing
- ◯ Patient support
- ◯ Other

If other, please specify.

____

How many years have you worked in industry?

____

Prior to working in an industry position, how many years did you work with patients with IEM in a clinical setting?

- ◯ N/A, have not worked in IEM clinically
- ◯ 1-5 years
- ◯ 6-10 years
- ◯ 11-15 years
- ◯ 16-20 years
- ◯ >20 years

What was your primary reason for choosing to move from a clinical position to an industry position?

____
(Optional)

Do you primarily interact with patients or health care providers?	◯ Patients<br>◯ Health care providers<br>◯ Both<br>◯ Little interaction with either group
Do you directly supervise any employees in your current position?	◯ Yes<br>◯ No
How many people do you supervise?	__________________________________
What annual budget size are you responsible for managing?	◯ None/Not applicable<br>◯ Less than $50K<br>◯ $50-99K<br>◯ $100-249K<br>◯ $250-499K<br>◯ $500K+
In your current position, what percentage of time are you assigned to specifically work with IEM?	__________________________________<br>(% of time)
What percentage of your time was spent working remotely over the last year?	◯ 0 to 24%<br>◯ 25 to 49%<br>◯ 50 to 74%<br>◯ 75 to 99%<br>◯ 100%
Please specify how you bill for your clinical nutrition services.	◯ Bill in my name only<br>◯ Bill in my name and MD<br>◯ Comprehensive fee for an interdisciplinary team visit<br>◯ Contractual/per diem<br>◯ Facility Fee<br>◯ Do not bill for services<br>◯ Unsure
Which type(s) of funds cover your salary? Please select all that apply.	☐ Clinic/hospital funds<br>☐ Clinical research grants<br>☐ Newborn screening grants<br>☐ Other grants<br>☐ Other (please specify)<br>☐ Unsure
If other, please specify.	__________________________________

List all of the professional activities that you have been involved in within the last 5 years. Check all that apply.

- ☐ Served on board or committee for professional organization(s), patient organization(s), industry program(s), and/or state/national government program(s)
- ☐ Developed or organized a conference, workshop, or symposium for health professionals
- ☐ Presented lecture or poster presentation at conference for health professionals and/or patient organizations
- ☐ Taught university-level course(s)
- ☐ Authored article(s) published in peer-reviewed journal(s), book(s) and/or book chapter(s)
- ☐ Authored grant proposal(s)
- ☐ Served as a peer-reviewer and/or on an editorial board for a journal
- ☐ Other
- ☐ None

Faculty

Do you have a faculty appointment?	◯ Yes<br>◯ No
What type of faculty appointment?	◯ Adjunct faculty<br>◯ Instructor/Lecturer<br>◯ Assistant Professor<br>◯ Associate Professor<br>◯ Professor<br>◯ Clinical Instructor/Lecturer<br>◯ Clinical Assistant Professor<br>◯ Clinical Associate Professor<br>◯ Clinical Professor<br>◯ Other title
Please select one of the following that best describes your faculty appointment.	◯ Tenured<br>◯ Tenure track<br>◯ Not tenure track
When you received your faculty appointment, what percentage salary increase did you receive?	◯ < 10%<br>◯ 10-19%<br>◯ 20-29%<br>◯ 30-39%<br>◯ 40-49%<br>◯ 50+%<br>◯ No increase in salary<br>◯ I started as faculty - not applicable

Salary and Benefits

What is your gross annual salary or wage in your current position? Please report in US dollars.

____
(Write as whole number (e.g., 65000))

On how many hours per week is that salary based? Please report standard hours, not extra hours you may have actually worked.

____
(Hours/week (Numbers only))

What is your salary classification?

- ◯ Salaried or exempt employee
- ◯ Hourly or non-exempt employee
- ◯ Private practice

What type of overtime compensation are you provided?

- ◯ Financially (additional pay/bonus/incentives)
- ◯ Compensatory time off work
- ◯ No compensation
- ◯ Unsure

In your current position, did your salary or hourly pay change in 2023?

- ◯ No change
- ◯ Increase in salary
- ◯ Temporary decrease in salary
- ◯ Permanent decrease in salary
- ◯ N/A, started a new position in 2023

Why did your pay increase in 2023?

- ☐ Cost-of-living adjustment
- ☐ Merit based
- ☐ Promotion
- ☐ Pay scale increase (market adjustment)
- ☐ Other
- ☐ Unsure

Approximately what percentage increase in salary/hourly pay did you receive in 2023?

____
(% increase)

Did you actively attempt to increase your salary over the last year (e.g., discuss with your supervisor, apply for a promotion, etc.)?

- ◯ No
- ◯ Yes, and attempt was fully successful
- ◯ Yes, and attempt was partially successful
- ◯ Yes, and attempt was unsuccessful

Does your current position provide benefits (e.g., insurance, retirement, paid time off, etc.)?

- ◯ Yes
- ◯ No

What benefits are included in your current benefit package plan (whether you took advantage of them or not). List all that apply.

	EMPLOYER OFFERS AND CONTRIBUTES	EMPLOYER OFFERS BUT DOES NOT CONTRIBUTE	NOT OFFERED	DON'T KNOW
401 K/retirement plan	◯	◯	◯	◯
403b plan	◯	◯	◯	◯
Pension plan	◯	◯	◯	◯
Health insurance/national health care	◯	◯	◯	◯
Dental insurance	◯	◯	◯	◯
Vision plan	◯	◯	◯	◯
Maternity and/or paternity leave (including paid and unpaid)	◯	◯	◯	◯
Disability (short and/or long term)	◯	◯	◯	◯
Life insurance	◯	◯	◯	◯
Stock options	◯	◯	◯	◯
Profit sharing	◯	◯	◯	◯
Pre-tax reimbursement programs (childcare, medical, etc.)	◯	◯	◯	◯
Tuition reimbursement	◯	◯	◯	◯
Student loan repayment	◯	◯	◯	◯
Wellness programs	◯	◯	◯	◯
Transportation/Parking	◯	◯	◯	◯

Does your employer provide paid time off on federal holidays?

- ◯ Yes, all 11 federal holidays
- ◯ Yes, some federal holidays
- ◯ No

How much personal time off (sick and vacation leave) are you given per year? Exclude holidays.

- ◯ < 10 days
- ◯ 10-14 days
- ◯ 15-19 days
- ◯ 20-24 days
- ◯ 25-29 days
- ◯ 30+ days
- ◯ Unlimited/flexible/discretionary PTO

Does unused personal time off that you accrue on an annual basis carry over to the next year?

- ◯ Yes
- ◯ No

Which of these other benefits were offered as part of your employment in your current position (whether you used them or not)? Please select all that apply.

- ☐ Working remotely (100% of working hours)
- ☐ Working remotely (< 100% of working hours)
- ☐ Flexible work schedule
- ☐ Paid and/or extended parental leave
- ☐ Paid time for professional development (e.g., conferences)
- ☐ Additional time off (e.g., two weeks off over Christmas break)
- ☐ Annual cash bonuses
- ☐ None of the above

Approximately how much money did you receive for your last annual cash bonus?	◯ Less than $250<br>◯ $250 to $499<br>◯ $500 to $999<br>◯ $1000 to $1499<br>◯ $1500 or more

Indicate the level of reimbursement from your current employer for the following work-related expenses.

	COMPLETE	PARTIAL	NONE	DON'T KNOW
Licensure fees	○	○	○	○
Professional association membership fees (GMDI, SIMD, SSIEM, etc.)	○	○	○	○
Conference registration fees	○	○	○	○
Travel expenses for conferences	○	○	○	○
Continuing education credits	○	○	○	○
Home office supplies (e.g., computer monitor, printer); do NOT include employer provided laptop	○	○	○	○
Books/journals	○	○	○	○
Specialty exams (e.g., CDR certification exams)	○	○	○	○

Are there any stipulations to access employer conference/travel funds? Select all that apply.

- ☐ Funding is dependent on annual budget
- ☐ Funding is dependent on speaking at a given conference
- ☐ Funding is only given some years (e.g., every other year)
- ☐ Attendance at some conferences is capped by my employer
- ☐ Funding is dependent on conference location
- ☐ Other reason

What is your annual conference budget allowance for 2023?

- ○ Based on annual division/departmental conference budget
- ○ Allotted 1 conference every other year regardless of budget
- ○ Allotted 1 conference per year regardless of budget
- ○ Allotted 2 conferences per year regardless of budget
- ○ Allotted more than 2 conference per year regardless of budget
- ○ Other
- ○ Unsure

If other, please specify.

____

Over the last year, what type of PAID professional activities did you participate in that are outside of your primary job? Check all that apply.

- ☐ Teaching
- ☐ Lectures/honoraria
- ☐ Consulting (e.g., AdBoards, speaking engagements)
- ☐ Private practice
- ☐ Writing
- ☐ Other
- ☐ None

If other, please specify.

____

Approximately how many paid, outside professional activities / separate contracts did you participate in over the last year?	______________________________<br>(Whole number (no ranges))
Over the last year, approximately how much total cash did you earn from paid, outside professional activities?	______________________________<br>(Write as whole number (e.g., 5000))
What are your top two primary reasons for participating in paid professional activities outside of your current job?<br>Please select your TOP TWO reasons only.	☐ Additional financial compensation<br>☐ Professional development<br>☐ Networking opportunities<br>☐ Opportunity to travel<br>☐ Opportunity to educate others about the nutritional management of IEM<br>☐ Other
If other, please specify.	______________________________________

Satisfaction with the Field of Inborn Errors of Metabolism

How satisfied are you with your professional focus in IEM?

- ◯ Unsatisfied
- ◯ Somewhat satisfied
- ◯ Moderately satisfied
- ◯ Very satisfied

How satisfied are you with the following aspects of working in the area of IEM at your facility?

	EXTREMELY DISSATISFIED	DISSATISFIED	NEUTRAL	SATISFIED	EXTREMELY SATISFIED	N/A
Scientific rigor	○	○	○	○	○	○
Patient relations	○	○	○	○	○	○
Respect from medical community	○	○	○	○	○	○
Professional growth/career advancement	○	○	○	○	○	○
Learning opportunities	○	○	○	○	○	○
Earnings potential	○	○	○	○	○	○
Research opportunities	○	○	○	○	○	○
Teaching opportunities	○	○	○	○	○	○

How can GMDI support your efforts for increased compensation and/or quality of life while working in the field of IEM? ________________________________

Is there anything that you want to share that provides pertinent information about your professional position and compensation that was not addressed by the questions included in this questionnaire? Please add your comments. ________________________________
