# Supplementary material for: Compensation for metabolic dietitians practicing in the United States: 2023 genetic metabolic dietitians international professional status survey
Source: Mol Genet Metab Rep. 2024 Sep 29;41:101147. doi: 10.1016/j.ymgmr.2024.101147 (PMC11470630; doi:10.1016/j.ymgmr.2024.101147)
Supplement: Supplementary material 2 [file mmc2.pdf]

Supplementary Tables 1-3

Supplementary Table 1. Characteristics of dietitians categorized as Level I, II, III, and IV dietitians according to facility career ladders.

	**Level I** n=8	**Level II** n=17	**Level III** n=15	**Level IV** n=9
**Years working as a RD, median (IQR)**	3 (ID)	12 (6-19)	11 (7-27)	19 (ID)
**Years in current job, median (IQR)**	1.1 (ID)	6 (4.75-12)	8 (4-21)	12 (ID)
**Years working with IEM, median (IQR)**	1 (ID)	6 (2-12)	5 (2-10)	13 (ID)
**Graduate degree, n(%)**	7 (88)	12 (71)	12 (80)	8 (89)
**Board certification, n(%)**	2 (25)	10 (59)	9 (60)	7 (78)
**Supervisor, n(%)**	0 (0)	0 (0)	2 (13)	4 (44)
**Faculty, n(%)**	0 (0)	0 (0)	1 (7)	1 (11)
**Other professional activities, n(%)**				
**Presented lecture or poster presentation at conference**	3 (38)	5 (29)	8 (53)	4 (44)
**Authored peer-reviewed article(s) and/or book chapter(s)**	3 (38)	3 (18)	5 (33)	5 (55)
**Served on board or committee due to professional expertise**	2 (25)	8 (47)	11 (73)	7 (78)
**Developed or organized a conference, workshop, and/or symposium**	0 (0)	3 (18)	4 (27)	3 (33)
**Taught university-level course(s)**	0 (0)	2 (12)	2 (13)	2 (22)
**Authored grant proposal(s)**	1 (13)	0 (0)	2 (13)	1 (11)
**Served as a peer-reviewer and/or on a journal's editorial board**	0 (0)	1 (6)	3 (20)	1 (11)

ID, insufficient data (subgroups with <10 respondents)

Supplementary Table 2. Percentage of respondents receiving benefits offered by their employer.

	Offers and contributes n (%)	Offers but does not contribute n (%)	Does not offer n (%)
**Health insurance**	110 (88)	14 (11)	1 (1)
**Dental insurance**	91 (73)	32 (26)	2 (2)
**Vision insurance**	85 (69)	35 (29)	2 (2)
**Disability insurance**	80 (65)	32 (26)	3 (2)
**Life insurance**	78 (62)	40 (32)	3 (2)
**401k retirement account**	76 (65)	2 (2)	33 (29)
**403b retirement account**	50 (43)	18 (15)	27 (23)
**Wellness programs**	61 (49)	30 (24)	17 (14)
**Transportation/Parking**	30 (24)	35 (28)	49 (39)
**Stock options**	9 (7)	8 (7)	82 (67)
**Profit sharing**	5 (4)	4 (3)	81 (67)

Supplementary Table 3. Respondent satisfaction with the following aspects of their current position.

	Extremely Satisfied n (%)	Satisfied n (%)	Neutral n (%)	Dissatisfied n (%)	Extremely Dissatisfied n (%)
**Patient relations**	59 (45)	58 (45)	3 (2)	3 (2)	0 (0)
**Scientific rigor**	58 (45)	52 (40)	11 (9)	3 (2)	1 (1)
**Learning opportunities**	51 (39)	56 (43)	14 (11)	4 (3)	1 (1)
**Respect from medical community**	43 (33)	64 (49)	15 (12)	4 (3)	1 (1)
**Research opportunities**	23 (18)	52 (40)	34 (26)	10 (8)	5 (4)
**Career advancement / Professional Development**	25 (19)	47 (36)	37 (29)	12 (9)	5 (4)
**Teaching opportunities**	11 (9)	42 (33)	47 (36)	13 (10)	2 (2)
**Earnings potential**	15 (12)	25 (19)	35 (27)	38 (29)	14 (11)
